# Optimising recruitment into trials using an internal pilot

**DOI:** 10.1186/s13063-019-3296-5

**Published:** 2019-04-11

**Authors:** W. Bertram, A. Moore, V. Wylde, R. Gooberman-Hill

**Affiliations:** 1 Musculoskeletal Research Unit, Translational Health Sciences, Bristol Medical School, University of Bristol, Level 1, Learning & Research Building, Southmead Hospital, Bristol, BS10 5NB UK; 2 North Bristol NHS Trust, Southmead Hospital, Bristol, BS10 5NB UK; 3 National Institute for Health Research Bristol Biomedical Research Centre, University Hospitals Bristol NHS Foundation Trust and University of Bristol, Bristol, UK

## Abstract

**Background:**

Recruitment to trials can be difficult. Despite careful planning and research that outlines ways to improve recruitment, many trials do not achieve their target on time and require extensions of funding or time.

**Methods:**

We describe a trial in which an internal pilot with embedded qualitative research was used to improve recruitment processes and inform recruitment projections for the main trial. At the end of the pilot, it was clear that the sample size would not be met on time. Three steps were taken to optimise recruitment: (1) adjustments were made to the recruitment process using information from the qualitative work done in the pilot and advice from a patient and public involvement group, (2) additional recruiting sites were included based on site feasibility assessments and (3) a projection equation was used to estimate recruitment at each site and overall trial recruitment.

**Results:**

Qualitative work during the pilot phase allowed us to develop strategies to optimise recruitment during the main trial, which were incorporated into patient information packs, the standard operating procedures and training sessions with recruiters. From our experience of feasibility assessments, we developed a checklist of recommended considerations for feasibility assessments. For recruitment projections, we developed a four-stage projection equation that estimates the number of participants recruited using a conversion rate of the number randomised divided by the number screened.

**Conclusions:**

This work provides recommendations for feasibility assessments and an easy-to-use projection tool, which can be applied to other trials to help ensure they reach the required sample size.

**Trial registration:**

ISRCTN, ISRCTN92545361. Registered on 6 September 2016.

**Electronic supplementary material:**

The online version of this article (10.1186/s13063-019-3296-5) contains supplementary material, which is available to authorized users.

## Background

When designing trials, good estimates of recruitment targets are needed. This enables study timeframes and budgets to be properly calculated and enables practicalities, such as the number of study centres, to be planned for. Despite careful planning, many trials struggle to achieve their recruitment targets within the expected timeframe. In a survey of UK clinical trial units, this was found to be the most commonly listed inefficiency in trials [1]. Numerous trials have stopped because they have not been able to recruit sufficient participants or have finished with fewer participants than planned [2]. Extensions of time and funding are required in around half of publicly funded trials [3–5]. In recent years, research has addressed trial recruitment, which has led to recommendations of strategies that may influence or improve recruitment, such as telephone reminders, financial and non-monetary incentives, envelopes with handwritten addresses, addressing external influences, using a patient-centred approach, making interventions available only in the trial and a dedicated trial manager [3, 6–10]. Despite this, questions remain unanswered about how to improve recruitment [11, 12].

Many authors have published on the difficulties in recruiting patients to trials and achieving complete samples [13]. Recruitment targets are often overly optimistic. Lasagna’s law—developed based on a trial in which only 100 patients were recruited from a potentially eligible population of over 8000 [14]—states that investigators overestimate the number of patients who may be available for a trial. The same phenomenon is described in Meunch’s third law, which states that any recruitment estimate should be divided by ten [15]. Despite much work that explores how to enhance recruitment, Lasagna’s law still holds true [16]. Less attention has been paid to how to estimate realistic recruitment rates in the first instance. Pilot phases (either internal or external) in trials are of particular value to the estimation of recruitment rates and strategies and are part of good trial design and practice [17, 18]. Internal pilots are useful for bringing unexpected practicalities to light that may be seen only once a trial is in the delivery phase.

Because of recruitment challenges, trial teams often need to expand to more centres or seek a time or funding extension to facilitate recruitment to target, while others do not recruit enough patients to answer their question. This is an inefficient use of resources, including time, money and effort for patients, researchers and funders [19, 20]. The key to achieving recruitment targets is to set them realistically by conducting a thorough feasibility assessment and using the information from this to create projections. This can be done using information from an internal pilot study. This article presents a case study of a randomised controlled trial in which we used information from an internal pilot phase to improve recruitment processes, undertake site feasibility assessments and create a projection tool to inform implementation of changes to the trial processes. Methods for predicting recruitment can be done using statistical packages [21, 22], but many researchers and trial managers do not have the training, time or resources to utilise them.

Our aim is to provide an overview of implementable methods of optimising recruitment, which research teams can use to increase the likelihood that recruitment targets are met.

### Case study: the STAR trial

This example describes the STAR trial, a randomised controlled trial that evaluated the clinical effectiveness and cost-effectiveness of a new care pathway for patients with chronic pain after a knee replacement. The trial was planned to run at four sites with a target sample size of 380. The full protocol has been published [23]. Recruitment was projected equally between centres with an estimated recruitment rate of 3 patients per month for all four sites combined during a 6-month pilot and 15 patients per month thereafter for a total of 30 months. Chronic pain after a knee replacement affects about 20% of patients, and therefore around 1 in 5 patients undergoing a total knee replacement would be eligible for the STAR trial [24–26]. Screening processes for the trial were piloted before the trial started and demonstrated a conversion rate of 7.6%, which is around 7–8 randomised patients per 100 screened [27]. The trial screening and recruitment processes are outlined in the flow chart in Additional file 1.

## Result

### Internal pilot

Internal pilots can help to identify unexpected recruitment issues. A 6-month internal pilot was run within the trial to refine recruitment procedures. This was undertaken following work with the same patient population to refine the intervention [27]; therefore, we did not conduct a separate feasibility study. At the completion of the pilot, the recruitment data were used to develop projections. These showed a predicted total of 166 randomisations at the end of the 30-month recruitment period. It was clear that the sample size of 380 would not be met on time. Three steps were undertaken to optimise recruitment following the pilot:

1. Adjustments were made to the recruitment process using information from qualitative work done in the pilot and advice from a patient and public involvement group.
2. Additional recruiting sites were included based on site feasibility assessments.
3. A projection equation was used to estimate recruitment at each site and overall trial recruitment.

### Step 1: embedded qualitative research

Recent research has demonstrated the benefit of using qualitative methods to inform refinements to trial processes and to optimise recruitment and retention rates [28, 29]. As part of the work within the pilot, we employed qualitative methods to identify and understand the motivations and possible barriers to participation of our target patient population. We used a dynamic approach commensurate with the trial design and the aims of the qualitative work [30], which were to identify barriers to recruitment and retention, and to optimise the accessibility of trial information and informed consent. Our approach involved audio-recording recruitment consultations between patients and recruiters, and follow-up telephone interviews with participants after they had been randomised.

Using purposive sampling [31] to ensure that data were elicited from patients and recruiters from a variety of trial sites, we aimed to record 30 recruitment consultations and conduct telephone follow-up interviews with 30 participants to explore their experience of trial recruitment and to identify facilitators and barriers to recruitment and retention, and the accessibility of trial information. The sample size was calculated as most likely to achieve data saturation [32]. During the recruitment consultation, patients were invited to participate in a follow-up telephone interview about their experiences of the recruitment process and randomisation. These interviews were conducted by an experienced qualitative researcher not previously known to the participants (AM) and audio-recorded with the participants’ written consent. Altogether, 31 recruitment consultations were audio-recorded, lasting a mean of 25 min, and 29 telephone interviews conducted, lasting a mean average of 17 min. Transcripts were coded and analysed using a thematic approach [33]. All our interpretations are based on an analysis of the transcribed data. Findings relevant to patient understanding of trial processes and information, illustrative data and actions are provided in Additional file 2. In summary:

- Some patients thought that the randomisation result reflected a calculated ‘need’ for the intervention based on their responses to the questionnaire. Further analysis showed that recruiters explained the use of questionnaires and entry of responses into a database just prior to explaining randomisation, so the link appeared natural.
- During the recruitment consultations, one patient was unsure about the term ‘ongoing treatment’ in reference to a statement in the patient information leaflet (PIL) that patients’ ongoing treatment would not be affected.
- One participant thought that they would be attending the clinic on multiple occasions as part of a group, which concerned them because of work commitments.
- Some participants experienced problems when selecting answers to questions in the online version of the outcome questionnaire.

Based on this information, the project team made the following changes to the recruitment process, PIL and questionnaires. We set up a working group to develop an improved explanation in the PIL of randomisation and the need for fair comparisons in trials. Moreover, we updated the standard operating procedures for recruiters to ensure that they made a clear distinction between completion of the questionnaire and the randomisation procedure. We expanded the description of usual care provided in the PIL to ensure that patients understood that all participants, regardless of group allocation, would still be able to access medical care as usual. We clarified in the PIL that patients would attend only one clinic appointment. Finally, the online version of the questionnaire was modified to ensure that participants could select options more easily.

During training sessions with recruiters, findings from the qualitative work were presented and discussed, ensuring that recruiters felt confident and supported during the main trial phase, and regular contact was maintained thereafter. Regular reports from the qualitative findings were also presented at trial management group meetings, steering committee meetings and patient and public involvement meetings, for discussion and reflection. The qualitative work helped us to develop strategies to optimise recruitment during the main trial.

#### Patient and public involvement

We collaborated with a study-specific patient and public involvement group to seek advice on patient documents and how to improve recruitment and retention methods [34]. The group met on five occasions before the trial and offered advice on the content and format of the plain English summary, PILs, questionnaires, resource-use diaries and interview topic guides. The group met three times during the pilot to review trial progress and give advice on standard operating procedures. Advice given included that research staff making phone calls or home visits should give patients plenty of time to answer the telephone or door as a painful knee can make getting up to walk difficult, especially after sitting for a long time. At the end of the pilot, we reviewed the findings with the group, who made recommendations about patient involvement in researcher training and updates to recruitment standard operating procedures, including how to explain usual care. The group met regularly throughout the trial and will give advice on how to communicate findings to patients and the public.

### Step 2: site feasibility assessments

A feasibility assessment is an evaluation of whether there is capacity and capability for a trial to be carried out at a site or group of sites. This trial management process is distinctly different from a feasibility study [30]. It is widely used in commercial research when selecting participating sites for clinical trials and in National Health Service (NHS) research departments in the United Kingdom when deciding whether there is capacity and capability to undertake delivery of a trial as a recruiting site. The process may help to identify barriers to recruitment and establish realistic recruitment targets and timelines [35]. An overview of the process and its importance is covered in the National Institute for Health Research (NIHR) good clinical practice training, which focuses on four considerations: patient population, study team, clinical support services, and equipment and facilities [36]. It is also important to consider external factors that may influence recruitment, such as changes in clinical guidelines or practices [8]. Assessing feasibility after a trial has opened can be useful in identifying issues with recruitment and informing the selection of additional sites. The key aspects of our feasibility assessments are described below.

Here is a summary of the recommended considerations for feasibility assessments:

- Patient population
  - Gain accurate estimates of how many patients are available.
  - Consider patient burden when estimating recruitment rates.
  - Consult a patient and public involvement group.
  - Embed qualitative research within the internal pilot.
  - Consider regional differences in patient populations.
- Study sites
  - Use multiple methods to identify sites, e.g. registries, local research departments and networks.
  - Send an expression of interest form to potential sites to collect feasibility information.
  - Expression of interest forms should ask for the following information:
    - ○ The trial team: training and experience, protected time available, cover arrangements
    - ○ A description of the current standard practice or treatment
    - ○ How the current standard differs from or conflicts with the trial intervention
    - ○ The number of potentially eligible patents and how this number was calculated
    - ○ Whether all (or some) clinicians support the recruitment of their patients
    - ○ Why some clinicians may not want to be involved
    - ○ Any conflicting trials currently running or being set up
    - ○ The number of trials the principal investigator is currently overseeing
    - ○ Whether support is available for additional costs
- Training, equipment and materials
  - Consider how much training is required for staff, and whether this will be provided centrally or on site.
  - Plan well in advance to account for busy schedules.
  - Specify if any specialist equipment or testing is needed, and clarify how or if the site can accommodate this.
- Setup
  - Approval processes will differ between sites: be prepared for delays and ask for timelines.
  - Ask each site what they need to move forward and stay in regular contact.

#### Patient population

First, we estimated the patient population at each potential trial site by searching local hospital systems, requesting data from hospital informatics departments and consulting data published by the UK National Joint Registry. Where electronic information was not available, we manually counted entries from clinic or procedure lists. Next, we estimated the number of patients who may want to take part in the trial. Regional variations in recruitment between each site were discussed with local research teams, who gave advice based on their previous experiences with the same or similar patient populations. Populations at recruiting sites were assessed using regular screening log submissions.

#### Site selection

Information published by the UK National Joint Registry identified hospitals performing a high volume of total knee replacements. Local research and development departments and research networks were consulted to help identify contacts who could be sent expression of interest requests. These requests were sent to many potential centres with a brief (one page) summary of the trial, either directly or via an intermediate contact, such as a local research network. The information required to conduct a feasibility assessment for a new site was requested in the expression of interest questionnaire, which can be found in Additional file 3. A deadline was placed on the return of the questionnaire.

The expression of interest asked for the number of eligible patients and how this figure was obtained, for example from hospital informatics departments. At one high-volume potential site, only half the clinicians agreed to allow the participation of patients they had treated. Thus, since not all the clinicians on some sites may be willing for the patients under their care to be recruited, we adjusted the targets and projections. An additional site was required to make up for the lost screening population. We considered each clinician’s reasons for not including patients, as these may be the same at other sites. We also investigated whether other studies were being run involving the same patient population, so that we had a sense of whether there was a risk that patients might be overburdened or invited to take part in other studies that might preclude their involvement in the trial, or vice versa. This trial involved excess treatment costs. We found it was important to be transparent to sites about these costs as well as about payments.

### Step 3: projections

After the pilot, we found that it was important not simply to divide the required sample size by the number of participating sites and assume each site would recruit equally. Some sites had differing numbers of available patients and recruitment targets were, therefore, weighted accordingly. Setup times were also considered, including a time lag between the commencement of screening and enrolment of the first participant. We used projections to estimate the monthly and overall recruitment from each site and adjusted these as the trial progressed to account for higher or lower recruitment. Our model used the estimated total number of participants to predict recruitment using a conversion rate of the number randomised divided by the number screened. The four-stage process is outlined below.

#### Stage 1: quantify the patient population

The Stage 1 metric is the total number of available potential participants to be screened in a given amount of time. We first clarified the different circumstances for each site. It was important to understand how their current clinical practice differs from the research intervention and how this might affect patient recruitment. Reliable information about the patient population was sourced from the National Joint Registry or electronic hospital databases to avoid using unrealistic estimates. Screening logs were submitted monthly by each recruiting site and were used to clarify the patient population. Stage one in the equation below represents the total number of patients available for screening across four sites (1456) in one year, or 12 months.

#### Stage 2: adjust for mitigating factors

The Stage 2 metric is the percentage of participants estimated to be eligible and willing to take part, or the conversion rate. We adjusted for known mitigating factors from the literature to calculate a potential conversion rate. After a systematic review, we thought it reasonable to assume that approximately 20% of patients with a total knee replacement would experience long term pain [24–26]. We then accounted for those patients deciding not to take part. Rather than set the conversion rate at 20%, we estimated that 50% of these patients would not meet the eligibility criteria and that 50% of those remaining would decline to take part, which leads to a potential conversion rate of 5%. The screening and randomisation logs can be used to calculate actual conversion rates by dividing the number of randomisations by the number screened. This can be used to project recruitment by site as well as the trial overall. Stage two in the equation below is 20% x 50% x 50%, or a conversion rate of 5%.

#### Stage 3: further adjustments

The Stage 3 metric is an adustment for expected losses to recruitment. Further adjustments were made to account for time periods during which all specialities experience challenges in recruitment to research studies. For example, the end of year holiday period is known to present challenges. We assumed that December and January would each lose half the potential recruitment. Stage three in the equation below represents 11 full recruitment months out of 12 (11/12), or a loss of 1 month recruitment per year. 

#### Stage 4: project recruitment

The Stage 4 metric is the total recruitment period. Once we had estimated our screening population and conversion rate, we applied our projection equation. This gave us an idea of what overall recruitment would be if all sites started on day one and recruited for the full 30 months. Stage four in the equation below represents the total recruitment period of 30 months.

### Projection equation

$$ \mathrm{Projected}\ \mathrm{recruitment}=\mathrm{Stage}\ 1\times \mathrm{Stage}\ 2\times \mathrm{Stage}\ 3\times \mathrm{Number}\ \mathrm{of}\ \mathrm{time}\ \mathrm{periods} $$

This equation was used to calculate the projected recruitment for our trial based on information from the original four sites after the internal pilot:

$$ \mathrm{Projected}\ \mathrm{recruitment}=\frac{1456}{12\ \mathrm{months}}\times \left(0.2\times 0.5\times 0.5\right)\times \left(\frac{11}{12}\right)\times 30\ \mathrm{months}=166.8 $$

This indicated that the current four sites in the pilot would not be sufficient to attain a sample size of 380. The information was then entered into a spreadsheet to project recruitment and estimate how many additional sites would be required to achieve the additional 214 randomisations, based on the patient population. Projections for each potential site were calculated to take into account the impact on overall trial recruitment. Five new sites were opened using a staged approach over time.

#### Trial delivery stage

Monthly screening log returns were used to calculate actual conversion rates. These were also used to observe whether sites were able to screen the agreed number of patients. When targets were not met, we worked with the site team to investigate ways to increase screening numbers. For example, we asked if there were particular operating lists or surgeons that were being missed or whether screening searches were being focused on a particular geographic area. Projections were then adjusted based on actual recruitment and conversion rate data. Actual and estimated data were included in the same projection by using actual data on current sites and projected data based on the trial average and a potential new site’s screening population.

**Fig. 1:**
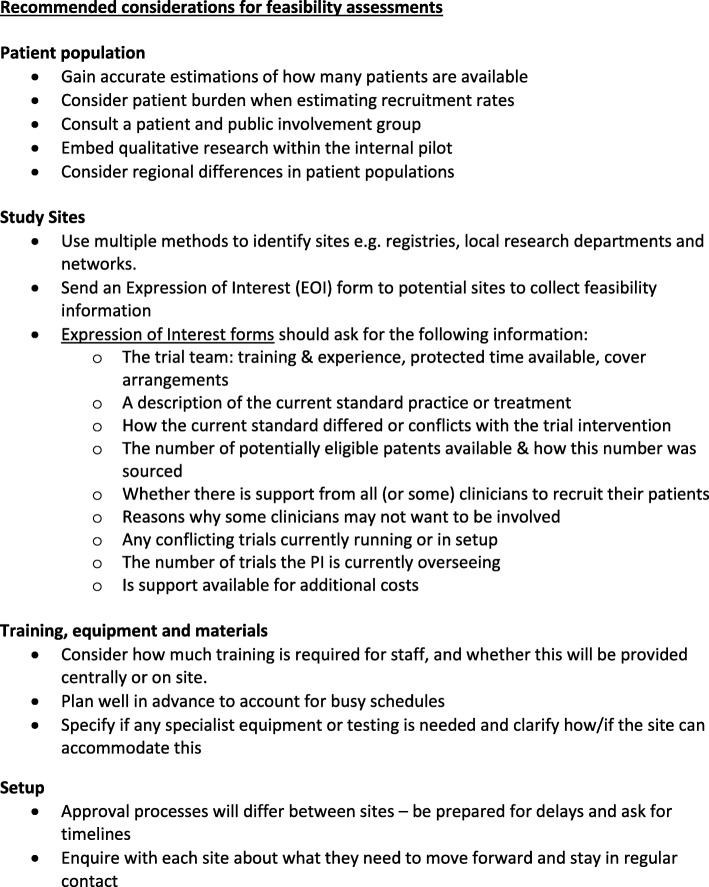
Example projection chart estimating recruitment with the addition of four potential sites opened in succession

These projections were used to estimate when the sample size would be reached and how many randomisations could be expected from recruiting sites, both current and potential, and by month and overall. This informed our decision-making on how many further sites would be required. Figure 1 shows an example projection created from month 7 to estimate recruitment compared to the target after opening four potential sites in succession. The formulas used to create these projections can be found in Additional file 4.

## Discussion

Feasibility assessments and recruitment projections are useful tools in designing a trial. Combined with an internal pilot and qualitative work, they allow trial management teams to set realistic recruitment targets and identify ways to improve recruitment, increasing the likelihood that the sample size is met. Using these tools during the pilot of a trial can optimise recruitment into the main trial and ensure delivery on time. Our internal pilot and the development of easy-to-use site feasibility and projection tools have helped to improve recruitment for the main trial, and key learning points can be applied to other trials.

The feasibility assessment that we have described is specific to the context of our trial; however, a similar approach could be applied to other trials with appropriate modifications. The main points to consider as outlined by the NIHR good clinical practice training are the patient population, trial staffing and training, clinical support services, and equipment and facilities. After the feasibility data have been collected and the sites confirmed, the setup is important to consider when setting projections of realistic targets.

Adjusting projections based on screening data is contingent on the submission of accurate screening logs. It has been argued that, despite the Consolidated Standards of Reporting Trials (CONSORT) guidelines, it is not worth collecting screening data [37], while others report the necessity of collecting screening data to comply with CONSORT guidelines and to report on the generalisability of the trial results [38, 39]. When published, screening data are not only important for transparency, but are also helpful for estimating the patient population in future trials. Screening data collected within a trial are valuable information, which may be used to project future recruitment.

Although projections are a useful tool for predicting recruitment, unexpected events will occur. We would suggest that these can be usefully viewed as a learning experience. Following the pilot, we learned to set recruitment to zero for the first month at new sites to give the team time to adjust to the trial procedures as well as to allow for a lengthy screening process. Adjustments for circumstances related to specific trials are important, especially when accounting for conditions that are affected by the time of year. Our projection tool enables the clear identification of the number of patients that could be randomised at each site and whether further study sites may be required. Our projection equation can be used to account for different variables, depending on the needs of the trial. To estimate the time required to recruit a sample, the equation is simply rearranged to divide the sample size by the results of stages 1–3:

$$ \mathrm{Numer}\ \mathrm{of}\ \mathrm{time}\ \mathrm{periods}=\frac{\mathrm{Sample}\ \mathrm{size}}{\mathrm{Stage}\ 1\times \mathrm{Stage}\ 2\times \mathrm{Stage}\ 3} $$

Although the methods described are specific to one trial, the concepts can be applied to many trials, both in the delivery phase and design phase. Information obtained from an internal pilot, including qualitative findings, can be used for site feasibility assessments and to create recruitment projections. This information may be used to improve recruitment procedures and processes and can inform a decision on whether to expand to additional trial sites. Use of these tools may help trial teams increase the likelihood that the sample size will be met.

## Additional files

Additional file 1: STAR trial screening and recruitment process flow chart. (DOCX 42 kb)

Additional file 2: Findings relevant to patient understanding of trial processes and information, illustrative data and actions. (DOCX 19 kb)

Additional file 3: STAR trial expression of interest questionnaire. (PDF 114 kb)

Additional file 4: Example of Excel formulas used to create projection tables. (JPG 172 kb)

## Abbreviations

CONSORT: Consolidated Standards of Reporting Trials
NHS: National Health Service
NIHR: National Institute for Health Research
PIL: Patient information leaflet

## References

[1] Duley L, Gillman A, Duggan M, Belson S, Knox J, McDonald A (2018). What are the main inefficiencies in trial conduct: a survey of UKCRC registered clinical trials units in the UK. Trials.

[2] Carlisle B, Kimmelman J, Ramsay T, MacKinnon N (2015). Unsuccessful trial accrual and human subjects protections: an empirical analysis of recently closed trials. Clin Trials.

[3] Walters SJ, Bonacho Dos Anjos Henriques-Cadby I, Bortolami O, Flight L, Hind D, Jacques RM (2017). Recruitment and retention of participants in randomised controlled trials: a review of trials funded and published by the United Kingdom Health Technology Assessment Programme. BMJ Open.

[4] Sully BGO, Julious SA, Nicholl J (2013). A reinvestigation of recruitment to randomised, controlled, multicenter trials: a review of trials funded by two UK funding agencies. Trials.

[5] Chapman SJ, Shelton B, Mahmood H, Fitzgerald JE, Harrison EM, Bhangu A (2014). Discontinuation and non-publication of surgical randomised controlled trials: observational study. BMJ.

[6] Treweek Shaun, Lockhart Pauline, Pitkethly Marie, Cook Jonathan A, Kjeldstrøm Monica, Johansen Marit, Taskila Taina K, Sullivan Frank M, Wilson Sue, Jackson Catherine, Jones Ritu, Mitchell Elizabeth D (2013). Methods to improve recruitment to randomised controlled trials: Cochrane systematic review and meta-analysis. BMJ Open.

[7] Edwards PJ, Roberts I, Clarke MJ, Diguiseppi C, Wentz R, Kwan I, et al. Methods to increase response to postal and electronic questionnaires. Cochrane Database Syst Rev. 2009;(3):MR000008. 10.1002/14651858.MR000008.pub4 PMC8941848 19588449

[8] Gillan MG, Ross S, Gilbert FJ, Grant AM, O’Dwyer PJ (2000). Recruitment to multicentre trials: the impact of external influences. Health Bull (Edinb).

[9] Chhatre S, Jefferson A, Cook R, Meeker CR, Kim JH, Hartz KM (2018). Patient-centered recruitment and retention for a randomized controlled study. Trials.

[10] Campbell MK, Snowdon C, Francis D, Elbourne D, McDonald AM, Knight R (2007). Recruitment to randomised trials: strategies for trial enrollment and participation study. The STEPS study. Health Technol Assess.

[11] Healy P, Galvin S, Williamson PR, Treweek S, Whiting C, Maeso B (2018). Identifying trial recruitment uncertainties using a James Lind Alliance Priority Setting Partnership - the PRioRiTy (Prioritising Recruitment in Randomised Trials) study. Trials.

[12] Gardner HR, Fraser C, MacLennan G, Treweek S (2016). A protocol for a systematic review of non-randomised evaluations of strategies to improve participant recruitment to randomised controlled trials. Syst Rev.

[13] Quick AM, Khaw PT, Elkington AR (1989). Problems encountered in recruiting patients to an ophthalmic drug trial. Br J Ophthalmol.

[14] Lasagna L (1979). Problems in publication of clinical trial methodology. Clin Pharmacol Ther.

[15] Bearman JE, Loewenson RB, Gullen WH (1974). Muensch’s Postulates, Laws and Corollaries.

[16] van der Wouden JC, Blankenstein AH, Huibers MJ, van der Windt DA, Stalman WA, Verhagen AP (2007). Survey among 78 studies showed that Lasagna’s law holds in Dutch primary care research. J Clin Epidemiol.

[17] Eldridge SM, Lancaster GA, Campbell MJ, Thabane L, Hopewell S, Coleman CL (2016). Defining Feasibility and Pilot Studies in Preparation for Randomised Controlled Trials: Development of a Conceptual Framework. PLoS One.

[18] White D, Hind D. Projection of participant recruitment to primary care research: a qualitative study. Trials. 2015;16:473. 10.1186/s13063-015-1002-9 PMC4615323 26482231

[19] Yordanov Y, Dechartres A, Porcher R, Boutron I, Altman DG, Ravaud P (2015). Avoidable waste of research related to inadequate methods in clinical trials. BMJ.

[20] Briel M, Olu KK, von Elm E, Kasenda B, Alturki R, Agarwal A (2016). A systematic review of discontinued trials suggested that most reasons for recruitment failure were preventable. J Clin Epidemiol.

[21] Gajewski BJ, Simon SD, Carlson SE (2012). On the Existence of Constant Accrual Rates in Clinical Trials and Direction for Future Research. Int J Stat Probab.

[22] Jiang Y, Guarino P, Ma S, Simon S, Mayo MS, Raghavan R (2016). Bayesian accrual prediction for interim review of clinical studies: open source R package and smartphone application. Trials.

[23] Wylde V, Bertram W, Beswick AD, Blom AW, Bruce J, Burston A (2018). Clinical- and cost-effectiveness of the STAR care pathway compared to usual care for patients with chronic pain after total knee replacement: study protocol for a UK randomised controlled trial. Trials.

[24] Beswick AD, Wylde V, Gooberman-Hill R, Blom A, Dieppe P (2012). What proportion of patients report long-term pain after total hip or knee replacement for osteoarthritis? A systematic review of prospective studies in unselected patients. BMJ Open.

[25] Pinedo-Villanueva R, Khalid S, Wylde V, Gooberman-Hill R, Soni A, Judge A (2018). Identifying individuals with chronic pain after knee replacement: a population-cohort, cluster-analysis of Oxford knee scores in 128,145 patients from the English National Health Service. BMC Musculoskelet Disord.

[26] Shim J, McLernon DJ, Hamilton D, Simpson HA, Beasley M, Macfarlane GJ (2018). Development of a clinical risk score for pain and function following total knee arthroplasty: results from the TRIO study. Rheumatol Adv Pract.

[27] Wylde V, Howells N, Bertram W, Moore AJ, Bruce J, McCabe C (2018). Development of a complex intervention for people with chronic pain after knee replacement: the STAR care pathway. Trials.

[28] Donovan JL, Rooshenas L, Jepson M, Elliott D, Wade J, Avery K (2016). Optimising recruitment and informed consent in randomised controlled trials: the development and implementation of the Quintet Recruitment Intervention (QRI). Trials.

[29] Elliott D, Husbands S, Hamdy FC, Holmberg L, Donovan JL (2017). Understanding and Improving Recruitment to Randomised Controlled Trials: Qualitative Research Approaches. Eur Urol.

[30] O’Cathain A, Hoddinott P, Lewin S, Thomas KJ, Young B, Adamson J (2015). Maximising the impact of qualitative research in feasibility studies for randomised controlled trials: guidance for researchers. Pilot Feasibility Stud.

[31] Robinson Oliver C. (2013). Sampling in Interview-Based Qualitative Research: A Theoretical and Practical Guide. Qualitative Research in Psychology.

[32] Guest Greg, Bunce Arwen, Johnson Laura (2006). How Many Interviews Are Enough?. Field Methods.

[33] Boyatzis RE (1998). Transforming qualitative information: Thematic analysis and code development.

[34] Bower P, Brueton V, Gamble C, Treweek S, Smith CT, Young B (2014). Interventions to improve recruitment and retention in clinical trials: a survey and workshop to assess current practice and future priorities. Trials.

[35] Johnson O (2015). An evidence-based approach to conducting clinical trial feasibility assessments. Clin Invest (Lond).

[36] Research NIfH. Introduction to GCP: National Institue for Health Research; [25/07/2018]. Available from: https://www.nihr.ac.uk/our-research-community/clinical-research-staff/learning-and-development/national-directory/good-clinical-practice/our-courses/introduction.htm. Accessed 4 Jan 2019.

[37] Elm JJ, Palesch Y, Easton JD, Lindblad A, Barsan W, Silbergleit R (2014). Screen failure data in clinical trials: Are screening logs worth it?. Clin Trials.

[38] Samaan Z, Thabane L (2014). Screen failure data in clinical trials: Are screening logs worth it? Commentary on ‘Elm et al’. Clin Trials.

[39] Slieker FJA, Kompanje EJO, Murray GD, Ohman J, Stocchetti N, Teasdale G (2008). Importance of screening logs in clinical trials for severe traumatic brain injury. Neurosurgery.

