# Supplementary material for: Optimising recruitment into trials using an internal pilot
Source: Trials. 2019 Apr 11;20:207. doi: 10.1186/s13063-019-3296-5 (PMC6458725; doi:10.1186/s13063-019-3296-5)
Supplement: Supplementary file 1 — STAR trial screening and recruitment process flow chart. (DOCX 42 kb) [file 13063_2019_3296_MOESM1_ESM.docx]

Additional file 1: STAR Trial screening and recruitment process.

Hospital records screened for potentially eligible patients

Patients posted a screening study pack to complete and return.

2 months

post-operative

Eligible patients identified from completed screening questionnaires and sent trial information pack.

All recruitment consultations recorded

+

29 participants interviewed

Eligibility checked with patient on the telephone. If eligible and willing to take part, recruitment consultation is scheduled.

Participants return completed baseline questionnaire.

3 months after surgery

Patients randomised.

3 months

post-operative
