# Supplementary material for: Optimising recruitment into trials using an internal pilot
Source: Trials. 2019 Apr 11;20:207. doi: 10.1186/s13063-019-3296-5 (PMC6458725; doi:10.1186/s13063-019-3296-5)
Supplement: Supplementary file 2 — Findings relevant to patient understanding of trial processes and information, illustrative data and actions. (DOCX 19 kb) [file 13063_2019_3296_MOESM2_ESM.docx]

Additional file 2

Findings from the qualitative work

| Qualitative Finding | Exemplary data | Action |
| --- | --- | --- |
| One participant was under the impression they would be attending the clinic on multiple occasions as part of a group, which concerned them because of work commitments. | Caroline: The only thing I thought of was when, erm, [recruiter] first said about attending a clinic. First of all, I had the impression if you were in the group that was going to a clinic, erm, that it could have been sort of weekly, fortnightly. I was sort of thinking about work, but she said no, it’s a one-off so, but that’s absolutely fine.  *Interviewer:* *Right, okay. Do you think that could be clearer in the information?*  Caroline: Erm, yes. Yeah, I think so. Yeah, I really had no idea how often that would be at all. I don’t think it mentioned it anywhere. | Additionally, we updated the patient information leaflet to emphasise that patients would only have to attend one clinic appointment |
| Some patients thought that the randomisation result reflected a calculated ‘need’ for the intervention based on their answers from their baseline questionnaire. Further analysis of these occurrences showed this to be a function of the timing of information exchange during the recruitment consultation; recruiters explained the use of questionnaires and entry of responses into a database just prior to explaining randomisation, so the link appeared natural. | Selma: I suppose in a way it would have been good to have participated in sort of follow up appointments, but I honestly felt that if I wasn’t needed, if there was enough people or I wasn’t sort of classed um... I don’t know it’s not a risk...um I can’t think of the word I'm thinking of [yeah] I obviously didn't meet the criteria to warrant it so obviously in my eyes that was good news.  *Interviewer: So I'm getting the sense that it was judged on some aspect of your condition if you like?*  Selma: Yeah because you know what I mean like I say this is not anything against you know because I work in a hospital and I know how important these trials are [yeah] yeah I suppose because it’s very hard you know the point of filling in the one to ten and this sort of thing you know I can only reflect on how I was at that point [yeah] um not anticipating what it was going to be like further down the line um…  Martin: I didn’t expect anything because, er, I sort of played down the pain I am getting, I’m not getting a great deal of pain but, er, I wouldn’t exaggerate it put it that way so I weren’t expecting to, er, get anything […]  *Interviewer: Right okay, that’s good to know, but you said – you said just that, er, because you don’t exaggerate the pain, your pain, then you were, you thought you might not get the intervention, so that sounds a little bit like you thought that that would be taken into account is that right?*  Martin: Well the more pain you’re getting the more you’ll get attention I would have thought. | We set up a working group to develop an explanation of randomisation and expanded the explanation of a need for fair comparisons in trials in the patient information leaflet and updated the Standard Operating Procedures for recruitment and provided further training to recruiters to give them confidence in explaining the need for fair comparisons in randomised controlled trials. |
| Some participants experienced problems when selecting answers to questions on the online version of the outcome questionnaire. | Well [laughs] [recruiter] did send me, erm, the, erm, you know, the, the, or at least the questionnaire over the, on the Internet and of course I work from my iPad because my husband has got the main computer and it’s a different email address for his invoice up there [yeah]. Erm, so but I can’t do ticks on the iPad. Some places you’ve got to circle, which I can’t do. Erm, some places you’ve got to tick and some places you’ve got to cross. Erm, [right] or I can do an x but I can’t tick you see [ah, okay]. So, and luckily enough [recruiter] had left me the paper copies as well so I actually then decided to fill that in and send it.  Ruth: I’m not really computer literate, it said to show where, does it say show where the pain still existed or something?  *Interviewer: Yes, it would have been a picture of a…*  Ruth: And I found that, virtually the last question I think it was and I couldn’t do it because erm I wasn’t quite sure how to do it [*Right, okay*] But that’s basically because I’m not computer literate really. […] I think it said something about label the part of it and I didn’t know how to do it. | The online version of the questionnaire was modified to ensure that participants could select options more easily. |
| During the recruitment consultations one patient was unsure about the term ‘ongoing treatment’ in reference to a statement in the patient information booklet that reassures patients that their ongoing treatment will not be affected. | The participant questioned the meaning of ‘ongoing treatment’ and suggested that they had not received any treatment following their operation.* | We expanded the description of usual care provided in the patient information leaflet to ensure that patients understand that regardless of treatment allocation all participants would still be able to access medical care as they normally would. |

*We did not request consent to publish direct quotations from the consultation interviews as we felt this would make recruiters and patients who had not yet agreed to participate in the study uncomfortable, so we have paraphrased as necessary.
