# Supplementary material for: Optimising recruitment into trials using an internal pilot
Source: Trials. 2019 Apr 11;20:207. doi: 10.1186/s13063-019-3296-5 (PMC6458725; doi:10.1186/s13063-019-3296-5)
Supplement: Supplementary file 3 — STAR trial expression of interest questionnaire. (PDF 114 kb) [file 13063_2019_3296_MOESM3_ESM.pdf]

Expression of Interest Questionnaire

Trial Site Team	
**Hospital Name & Trust**	
Principal Investigator	
PI Email & phone number	
PI mailing address	
Co-Investigator(s)	
Co- Inv Email & phone number	
Research Nurse/Physio/CTO	
Researcher email & phone number	
Researcher address	
Extended Scope Practitioner (ESP)	
ESP email & phone number	
ESP address	
R&D Contact	
R&D email & phone number	
R&D address	
Please list any additional staff who will be involved in the STAR trial at your site including contact details.	

Expression of Interest Questionnaire

Screening, Recruitment & Intervention	
How many consultants in your Trust perform total knee replacements?	
How many primary total knee replacements are carried out in your trust per year? Please provide the source of this number.	
What is the current standard care package in your trust for patients following primary total knee replacement?	
What methods will you use to screen patients who have had a total knee replacement for osteoarthritis? (e.g. theatre lists, IT systems)	
How many clinical research studies are currently active in your orthopaedic department?	
Are there any conflicting studies in set up or actively recruiting? Please list study titles.	
How many clinical research studies is the PI currently involved in? Please list study titles.	
How many clinical research studies is/are the researcher(s) working on? (Please include studies in set-up, recruitment and follow-up).	

University of BRISTOL

Star
Support and treatment after joint replacement

North Bristol NHS
NHS Trust

Expression of Interest Questionnaire

Excess Treatment Costs	
Do you have support from your R&D Department and the relevant clinical area to support excess treatment costs of up to £4000 per year? Please list the name and contact details of the relevant staff.	

Study Team	
This study involves home visits for recruitment consultations with potential participants. Are the local research team able to be mobile to perform home visits? *Please describe and include any experience the study team have undertaking home visits and any lone worker policies.*	
How much time (in WTE) is available for research staff to screen and recruit for STAR?	
Who is the named Orthopaedic Knee Consultant who will provide clinic oversight for the STAR Trial?	
Is an Extended Scope Practitioner (ESP) clinician available to deliver the STAR intervention and follow-up calls? This involves approximately 1-2 STAR clinics per month at up to four hours per clinic.	
Is the ESP willing to attend an intervention training day in Bristol? Travel expenses will be reimbursed.	

University of BRISTOL | Star — Support and treatment after joint replacement | North Bristol NHS Trust

Expression of Interest Questionnaire

Clinical Facilities

Is clinic space available for a twice monthly STAR clinic?	
Are facilities available to perform the required radiographs during STAR clinics?	
Are trained staff available in clinic to draw bloods?	

Space and Equipment:

Does the study team have dedicated separate room/office for clinical research	
Does the site have space for storage of study related materials?	
Does the site have space available for Sponsor monitoring visits?	
Does the site have space for Archival of data after completion of trial?	
Does the site have dedicated cupboard/filing cabinet for study document storage (CRFs, participant study folders)?	
Does the research team have a dedicated phone line which can be used as a point of contact for STAR participants?	
Does the research team have regular access to a scanner/photocopier?	

Details of Person completing Form:

Name	
Title	
Contact Details	
