# Supplementary figures and images for: Optimising recruitment into trials using an internal pilot
Source: Trials. 2019 Apr 11;20:207. doi: 10.1186/s13063-019-3296-5 (PMC6458725; doi:10.1186/s13063-019-3296-5)

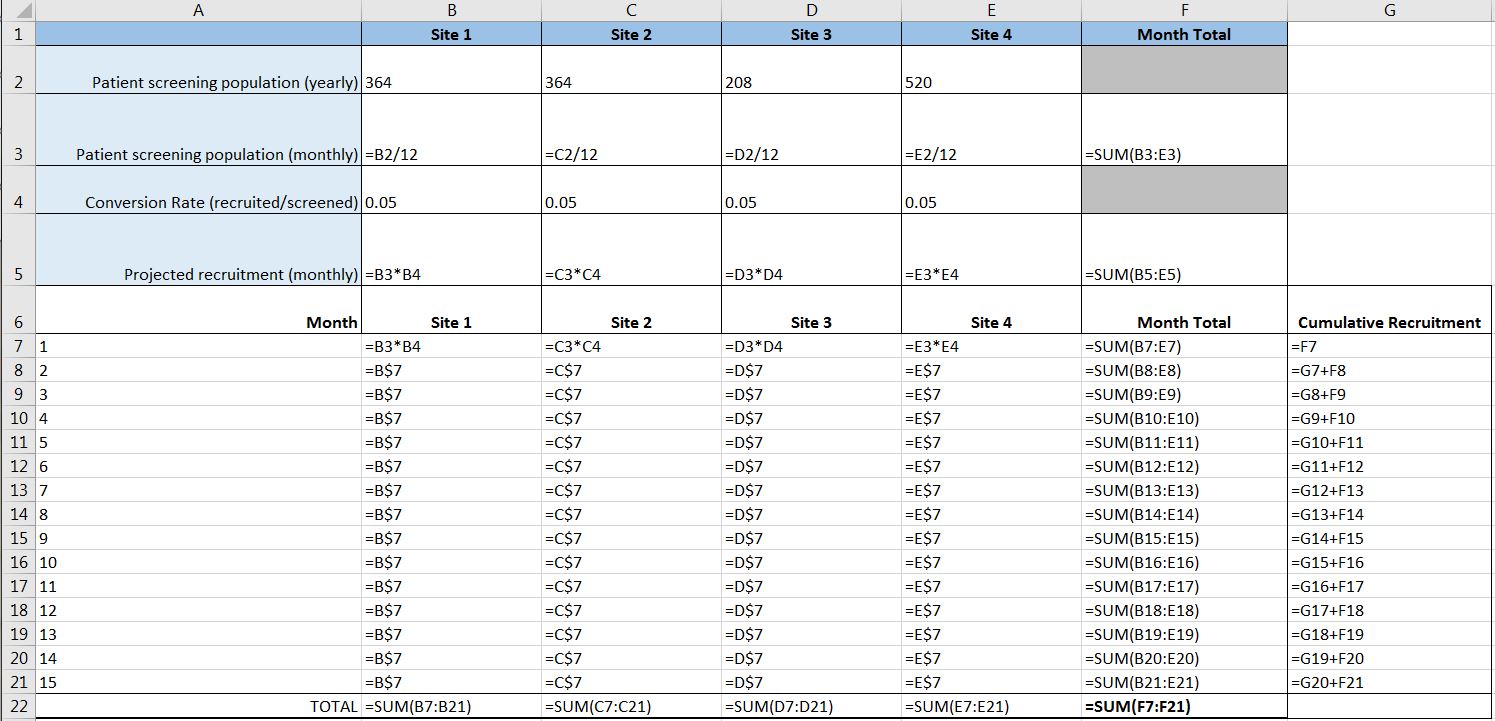

Supplement: Supplementary file 4 — Example of Excel formulas used to create projection tables. (JPG 172 kb) [file 13063_2019_3296_MOESM4_ESM.jpg]
